# Facial expression recognition and histograms of oriented gradients: a comprehensive study

**DOI:** 10.1186/s40064-015-1427-3

**Published:** 2015-10-26

**Authors:** Pierluigi Carcagnì, Marco Del Coco, Marco Leo, Cosimo Distante

**Affiliations:** National Research Council of Italy, Institute of Applied Sciences and Intelligent Systems, Via della Libertà, 3, 73010 Arnesano , LE Italy

**Keywords:** Facial expression recognition, HOG, SVM

## Abstract

Automatic facial expression recognition (FER) is a topic of growing interest mainly due to the rapid spread of assistive technology applications, as human–robot interaction, where a robust emotional awareness is a key point to best accomplish the assistive task. This paper proposes a comprehensive study on the application of histogram of oriented gradients (HOG) descriptor in the FER problem, highlighting as this powerful technique could be effectively exploited for this purpose. In particular, this paper highlights that a proper set of the HOG parameters can make this descriptor one of the most suitable to characterize facial expression peculiarities. A large experimental session, that can be divided into three different phases, was carried out exploiting a consolidated algorithmic pipeline. The first experimental phase was aimed at proving the suitability of the HOG descriptor to characterize facial expression traits and, to do this, a successful comparison with most commonly used FER frameworks was carried out. In the second experimental phase, different publicly available facial datasets were used to test the system on images acquired in different conditions (e.g. image resolution, lighting conditions, etc.). As a final phase, a test on continuous data streams was carried out on-line in order to validate the system in real-world operating conditions that simulated a real-time human–machine interaction.

## Background

Facial expression is one of the most common non-verbal way that humans use to convey internal emotional states and, consequentially, plays a fundamental role in interpersonal interactions. Although there exists a wide range of possible facial expressions, psychologists have identified the six basic ones (happiness, sadness, fear, disgust, surprise, and anger) that are universally recognized (Izard 1971). It is straightforward that a system capable to perform an automatic recognition of the human emotions is a desirable task for a set of emerging applications related to assistive technologies, digital signage, audience measurement and so on. More specifically, the capability to automatically recognize the emotional state of a human being is a key factor to the challenging field of the human–robot interaction since on the one hand it allows to introduce behavioral metrics and on the other hand it could increase the level of technology acceptance. Unfortunately, the design of a system with a high recognition rate is a non trivial task due to a wide set of problems. Some of the involved issues, such as scene illumination conditions (Song et al. 2010) and camera viewpoint (Lucey et al. 2010), can be addressed by selecting a proper acquisition set-up or by preprocessing operations. On the other hand, the variability in appearance of different subjects, and even of the same subject, performing the same expression introduces a bias factor that stresses the decision block of the FER system and that requires further investigation to be properly handled. Moreover, a robust FER system should deal with the intrinsic variations in performing the same expression for subjects of different ethnic and cultural backgrounds. For these reasons, in the last years, the interest of the computer vision community in FER has exponentially grown-up and a wide range of solutions can be found in literature. Proposed solutions can be divided into two main categories: the first category includes the solutions that classify human emotions by processing a set of consecutive images while, the second one, includes the approaches which perform FER on each single image. By working on image sequences much more information is available for the analysis. Usually, the neutral expression is used as a reference and some characteristics of facial traits are tracked over time in order to recognize the evolving expression (Dornaika et al. 2011). To this end, the use of key points and texture information  (Song et al. 2010), a modified version of the well known Local Binary Patterns (LBP) combined with moments (Ji and Idrissi 2012), a pyramid of LBP (Khan et al. 2013), a combination of Independent Component Analysis (ICA), Fisher Local Discriminant Analysis (FLDA) and Hidden Markow Models (HMM) (Uddin et al. 2009), optical flow and non-linear features (Siddiqi et al. 2014), are some of the most effective approaches used to represent facial traits to be tracked over time. The major drawback of these approaches is the inherent assumption that the sequence content evolves from the neutral expression to another one that has to be recognized. This constrain strongly limits their use in real world applications where the evolution of facial expressions is completely unpredictable. For this reason, the most attractive solutions are those performing facial expression recognition on a single image. The approaches in literature that work on a single image can be conveniently categorized depending on the strategies they use to lead towards the recognition of emotions. In this way, two main categories arise:

- Component based approaches
- Global approaches

Component based approaches preliminary extract some facial components and then try to classify expressions on the basis of the matching among corresponding components or by comparing the geometrical configuration among different components. An example is given by Pantic and Rothkrantz (2000) where an automatic facial expression system based on geometrical features is presented. The same authors in Pantic and Rothkrantz (2004) introduce a recognition system for facial expression analysis from static face images by exploiting ten profile-contour fiducial points and 19 fiducial points of the contours of the facial components. A comparison between geometric positions of a set of fiducial points and a set of multi-scale and multi-orientation Gabor wavelet coefficients is investigated in Zhang et al. (1998). Loconsole et al. (2014) propose the use of linear and eccentricity geometrical features in association with a machine learning classifier in order to maximize the discriminative capability and test their approaches with the recent Radboud Faces Database Langner et al. (2010). Poursaberi et al. investigate the use of Gauss–Laguerre wavelets in association with geometrical position of fiducial points in order to provide valuable information for the upper/lower face zone Poursaberi et al. (2012).

The work in Zhang and Tjondronegoro (2011) proposes the use of “salient” distance features, which are obtained by extracting patch-based 3D Gabor features, selecting the most discriminative patches and performing patch matching operations. More recently, Happy and Routray (2015) propose a novel framework for expression recognition by using appearance based features of selected facial patches depending on the position of facial landmarks that are active during emotion elicitation. Then patches are further processed to obtain the salient ones containing the most discriminative features for classification.

Unfortunately, although the idea of making use of a preliminary selection of salient facial components and a subsequent emotion recognition phase based on geometrical or textural matching has been widely investigated, the achieved classification performances do not fulfill the demanding requirements of the technologies that a FER system has to serve. The main unresolved issues concern the alignment of components in different facial images, especially in case of extreme expressions. Moreover, they experienced high computational time due to the load for the fine extraction of the facial components (especially when iterative strategies are used) and then they appear to be not suitable for real world applications especially if low-power systems are involved (e.g. assistive robot, consumer analysis devices) (Sadeghi et al. 2013).

The above mentioned problems can be overcome by using “Global Approaches” i.e. approaches that directly try to extract a representation of the expressions from the appearance of the global face. This research area has been deeply investigated, but there is still much effort to do since it is very challenging to find a global set of descriptors able to robustly characterize human expression traits. Some of the most recent related works, that have arisen as a consequence of the theoretical improvements in the definition of more reliable local descriptors, are listed below. In  Shan et al. (2009) authors use Local Binary Pattern (LBP) even in low resolution and compressed input images whereas, in Zhao and Zhang (2011), the same descriptors are combined with a kernel-based manifold learning method called Kernel Discriminant Isometric map (KDIsomap). Ramirez Rivera et al. (2013) propose in a new descriptor, named local directional number pattern (LDN), that extracts directional information by using compass masks and encodes such information using the prominent direction indices. The sparse representation-based classification (SRC) is used with a principal component analysis (PCA)-based dictionary in Mohammadi et al. (2014), in combination with the local phase quantization (LPQ) in Zhen and Zilu (2012) and Gabor filters in Liu et al. (2012). An algorithm for facial expression recognition, by integrating curvelet transform and online sequential extreme learning machine (OSELM) with radial basis function (RBF) hidden node, is proposed in Uçar et al. (2014).

As revealed in the previous discussion, FER problem is clearly related to the facial deformation. Different persons could express the same emotion with some differences, but the majority of the involved muscles work in such a way to give a coherent characterization of that emotions among different people of different ethnicity and gender.

This paper proposes an comprehensive study of the application of histogram of oriented gradients descriptor (HOG) in the FER problem highlighting as this powerful technique could be effectively exploited for this purposes. HOG is a shape descriptor that counts occurrences of gradient orientations in localized portions of an image and that is mainly used for the purpose of object detection but that is also intuitively useful to model the shape of the facial muscles by means of an edge analysis. To the best of our knowledge, HOG descriptor has been used as a tool for FER purposes in a few works. The use of a log-likelihood map in association with a hierarchical decision tree, in order to select the most discriminative HOG features in the HOG grid, is presented in Orrite et al. (2009) whereas the use of HOG and SVM is proposed in the context of a specific challenge in Dahmane and Meunier (2011) and successively in Chen et al. (2014); the first one uses an adaptive grid size whereas the second one focuses its novelty on the selection of sub-regions of interest. Anyway, in both cases, the descriptor is exploited with its standard parameters and the results are not completely satisfactory. HOG, jointly to sparse coding, is analyzed by OuYang and Sang (2013) in order to make the FER problem more robust respect to occlusions. The HOG descriptor is also used in conjunction with other descriptors. For instance it is associated to the Weber’s Local Descriptor (WLD) one in Wang et al. (2013). In Barroso et al. (2013) it is concatenated to LBP and SIFT (Scale-invariant feature transform) and a good analysis of the influence of the knowledge of the subject is done. The only work that performs a more accurate investigation of importance of parameters is  Gritti et al. (2008) where authors, anyway, focus their attention on the alignment perturbations for different descriptors [HOG, Local Ternary Pattern (LTP), LBP and Gabor filters] and demonstrate that the best FER performances are obtained using LBP. Anyway, all the above mentioned works do not perform any investigation about how the parameters of the HOG descriptor can affect the FER process and therefore it is not proved that HOG descriptor has been exploited at its best.

The study, carried out in this paper, highlights that a proper set of the HOG parameters can make this descriptor one of the most suitable to characterize facial expression peculiarities. This has been demonstrated by a large experimental session where, exploiting a consolidated algorithmic pipeline, results showed that, using HOG to characterize facial expression peculiarities, the experienced FER outcomes can outperform the most commonly used FER frameworks. The algorithmic pipeline takes as input a single frontal facial image and it performs a preliminary face detection and registration (Castrillón et al. 2007) (allowing the following procedural phase to be performed in a more effective way). HOG descriptor is then applied on the registered face and finally the classification is performed by means of a set of SVMs. Additional experimental sessions, carried out on different publicly available datasets, give evidence that the emerged HOG parameter configuration is not dependent on the input data since it can be left unchanged when the FER pipeline is applied on different sets of data assuring anyway very encouraging FER performances outcomes. As a final step, a test on continuous data streams has been carried out on-line in order to validate the findings of the study in real-world operating conditions that simulated a real human–machine interaction. In this experimental phase, a temporal window and a consistency rule have been introduced to handle the incoming video streams in real time.

The rest of the paper is organized as follows: in "Methodology overview" the proposed pipeline is detailed with an in-depth description of each operating step. "Experimental data setup" is aimed at the datasets description whereas in "Experimental results: phase 1" the optimization of the HOG parameters and the comparison of the optimized pipeline with alternative appearance descriptors and leading state of the art approaches are supplied and discussed. "Experimental results: phase 2" reports the FER performances on additional publicly available datasets and, finally, "Experimental results: phase 3" reports the evaluation of the proposed approach on video sequences. "Conclusions" concludes the paper.

## Methodology overview

**Fig. 1:**
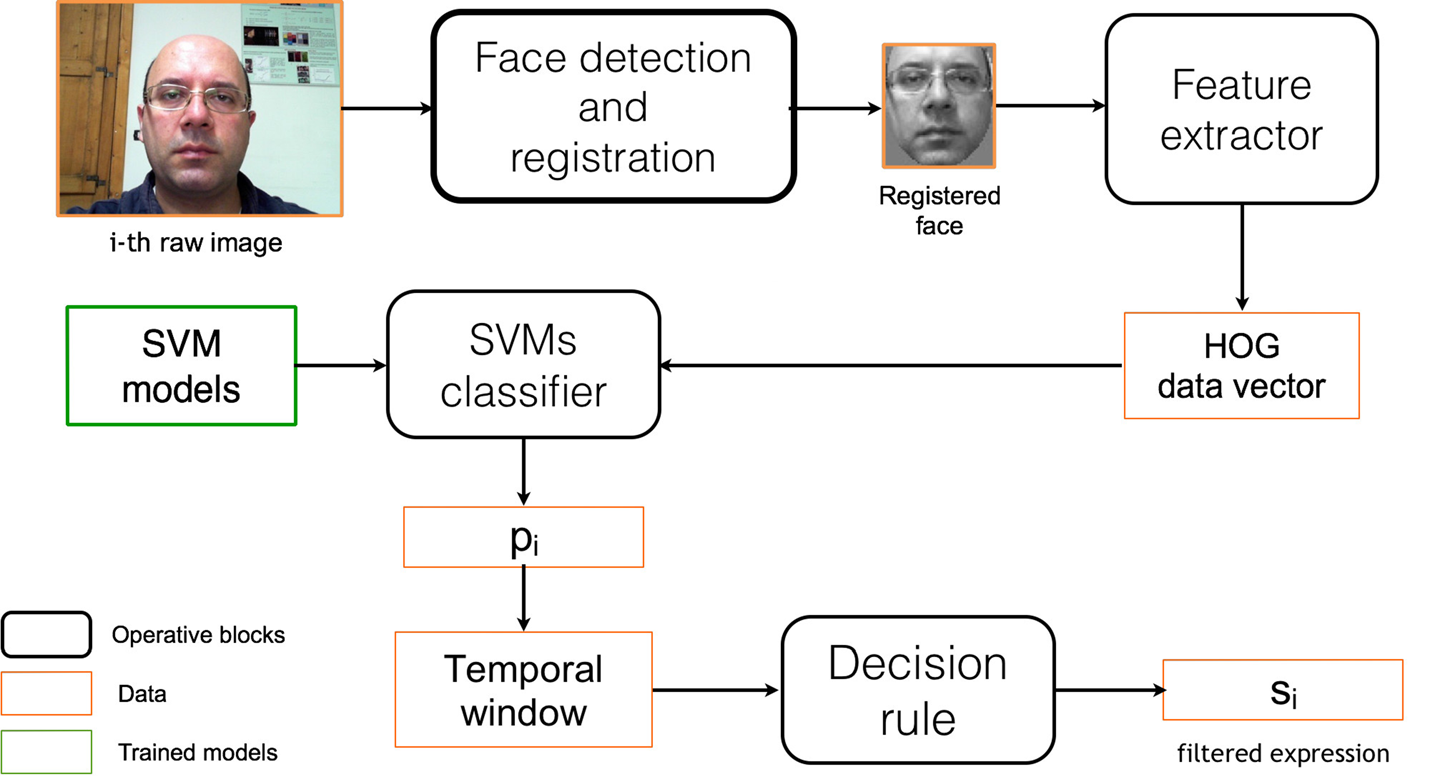
Proposed system pipeline: faces are cropped and registered and then HOG descriptor is applied to build a data vector that is provided as input to a SVM bank that gives the estimation of the observed facial expression. Finally, the prediction is queued in the temporal window exploited by the decision rule in order to filter possible misclassifications

Facial expression recognition, from generic images, requires an algorithmic pipeline that involves different operating blocks. The scheme in Fig. 1 has been used in this work: the first step detects human faces in the image under investigation and then detected faces are registered (Castrillón et al. 2007). This preliminary operations allow the system to get the quite similar position for the eyes and in this way the subsequent HOG descriptor may be applied using a coherent spatial reference. The vector of features extracted by HOG is finally used for the classification of the facial emotions by means of SVM strategies. Finally, the managing of the temporal images stream is demanded to an ad-hoc decision rule. Each operating step is detailed in the following subsections.

### Face detection and registration

**Fig. 2:**
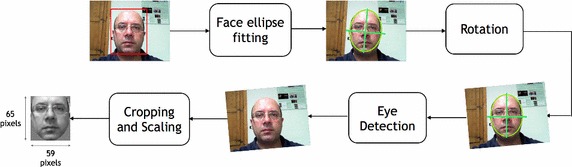
Face registration: the detected face is fitted in an ellipse used to rotate the face in a perfectly vertical position; successively eyes are detected and used to scale the image and to crop the area of interest

In this step, human faces are detected in the input images and then a registration operation is done. The registration is a fundamental preprocessing step since the subsequent algorithms work better if they can evaluate input faces with predefined size and pose. The face detection is performed by means of the general frontal face detector proposed by Viola and Jones (2004) which combines increasingly more complex classifiers in a cascade. Whenever a face is detected, the face registration is carried out as follows: the system, at first, fits an ellipse to the face blob (exploiting facial features color models) in order to rotate it to a vertical position and hence a Viola-Jones based eye detector searches the eyes. Finally, eye positions, if detected, provide a measure to crop and scale the frontal face candidate to a standard size of $65\times 59$ pixels. The above face registration procedure is schematized in Fig. 2.

The registered face is then modeled using different features (average color using red-green normalized color space and considering just the center of the estimated face container; eyes patterns; whole face pattern) in order to re-detect it, for tracking purposes, in the subsequent frames (Castrillón et al. 2007). Finally, it is given as input to the features extractor based on the HOG descriptor.

### HOG descriptor

**Fig. 3:**
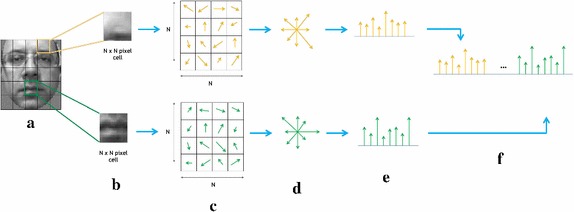
HOG features extraction process: image is divided in cells of size $N \times N$ pixels. The orientation of all pixels is computed and accumulated in an M-bins histogram of orientations. Finally, all cell histograms are concatenated in order to construct the final features vector. The example reports a cell size of 4 pixels and 8 orientation bins for the cell histograms

Local object appearance and shape can often be characterized rather well by the distribution of local intensity gradients or edge directions, even without precise knowledge of the corresponding gradient or edge positions. This statement leads to the definition of the HOG technique that has been used in its mature form in Scale Invariant Features Transformation (Lowe 2004) and it has been widely exploited in human detection (Dalal and Triggs 2005). HOG descriptor is based on the accumulation of gradient directions over the pixel of a small spatial region referred as “cell” and in the subsequent construction of a 1D histogram whose concatenation supplies the features vector to be considered for further purposes. Let *L* be an intensity (grayscale) function describing the image to be analysed. The image is divided into cells of size $N\times N$ pixels (as in Fig. 3a) and the orientation $\theta _{x,y}$ of the gradient in each pixel is computed (Fig. 3b, c) by means of the following rule:

1 $$\begin{aligned} \theta _{x,y}=\tan ^{-1}\frac{L(x,y+1)-L(x,y-1)}{L(x+1,y)-L(x-1,y)} \end{aligned}$$

Successively, the orientations $$\theta _i^j$$$$i=1 \ldots N^2$$, i.e. belonging to the same cell *j* are quantized and accumulated into a M-bins histogram (Fig. 3d, e). Finally, all the achieved histograms are ordered and concatenated into a unique HOG histogram (Fig. 3f) that is the final outcome of this algorithmic step, i.e. the features vector to be considered for the subsequent processing.

### SVM prediction

The HOG features vectors are then given as input to a group of Support Vector Machines (SVMs). SVM is a discriminative classifier defined by a separating hyperplane. Given a set of labelled training data (supervised learning), the algorithm computes an optimal hyperplane (the trained model) which categorizes new examples in the right class. Given the training vectors $\varvec{x}_i \in \mathbb {R}^n, i=1,\ldots ,l$ and the corresponding set of *l* labels $y_i \in \lbrace 1,-1 \rbrace$, the following primal optimization problem is solved:

$$\begin{aligned} \min _{\varvec{w}, b, \xi }&\quad \frac{1}{2}\varvec{w}^T\varvec{w}+C\sum _{i=1}^l \xi _i \\ \text {subject to }&\quad y_i(\varvec{w}^T \phi (\varvec{x}_i)+b)\ge 1-\xi _i, \\&\quad \xi _i\ge 0, i=1,\ldots ,l, \end{aligned}$$

where $\xi _i$ is the misclassification error for the *i*th training vector; $\xi$ is the total misclassification error; $\varvec{w}$ is the normal vector to the hyperplane; $b/\Vert \varvec{w}\Vert$ determines the offset of the hyperplane from the origin along the normal vector $\varvec{w}$ (with $\Vert \centerdot \Vert$ the norm operator); $\phi (\varvec{x}_i)$ maps $\varvec{x}_i$ into a higher-dimensional space and $C > 0$ is the regularization parameter.

Further theoretical notions about SVM, together with related implementation issues, can be found in Cortes and Vapnik (1995). The classical SVM approach is suitable only for a two classes problem but, unfortunately, FER involves multi-class handling. Multi-class problems can be addressed by the “one-against-one” method proposed in Knerr et al. (1990), an approach based on the construction of an SVM classifier for each pairwise of classes and a voting system aided to elect the predicted class when an unseen item is tested. More specifically, let *k* be the number of classes, then $k(k - 1)/2$ classifiers $K_{ij}$ are trained from the available data of the *i*th and *j*th classes. The final prediction is returned by a voting system: the unseen example is analysed by each classifier $K_{ij}$ that gives a vote to one or to the other class. Finally, the class with the largest number of votes is chosen. Although many other methods are available for multi-class SVM classification, in Hsu and Lin (2002) a detailed comparison is given with the conclusion that “one-against-one” is a competitive approach. In particular, the multi *C*-support vector classification (multi *C*-SVC) learning task implemented in the LIBSVM library (Chang and Lin 2011) was used in the experiments reported in "Experimental results: phase 1, Experimental results: phase 2 and Experimental results: phase 3". Radial Basis Function (RBF) was used as kernel as suggested in Hsu et al. (2003) for non-linearly separable problems with penalty parameter $C=1000$ and $\gamma =0.05$ .

### Temporal analysis

To make the system suitable for video sequence analysis, a decision making strategy based on the temporal consistency of FER outcomes has been introduced. The decision, about the expression in a video, is taken by analyzing a temporal window of size *m* and verifying if at least *n* ($n<m$) frames in the window are classified as containing the same facial expression. More specifically, the system performs a frame by frame analysis in the time window *w* of size *m* and for each frame an expression classification outcome is given as $p_i$ where *p* is the predicted expression and *i* is the current frame index.

The expression in the window is classified as the expression *s* if

2 $$\begin{aligned} \sum _{j=i-m+1}^{i}\left( \Lambda (p_j,s)\right) \ge n \end{aligned}$$

where $\Lambda (p_j,s)=1$ if $p_j=s$ and 0 otherwise.

This procedure allows the system to manage a temporal stream for a subject avoiding wrong expression predictions due to sporadic misclassifications.

## Experimental data setup

All the experimental sessions have been carried out on two publicly available datasets of image sequences specifically acquired for FER issues. The first dataset is the Chon-Kanade (CK+) Lucey et al. (2010): it is made up by image sequences of people performing 6 facial expressions. Each sequence starts with a neutral face expression and ends with the expressive face. The variety of subjects in terms of gender, as well as ethnicity and age, makes the dataset one of the most used to test the performance of FER solutions.

**Fig. 4:**
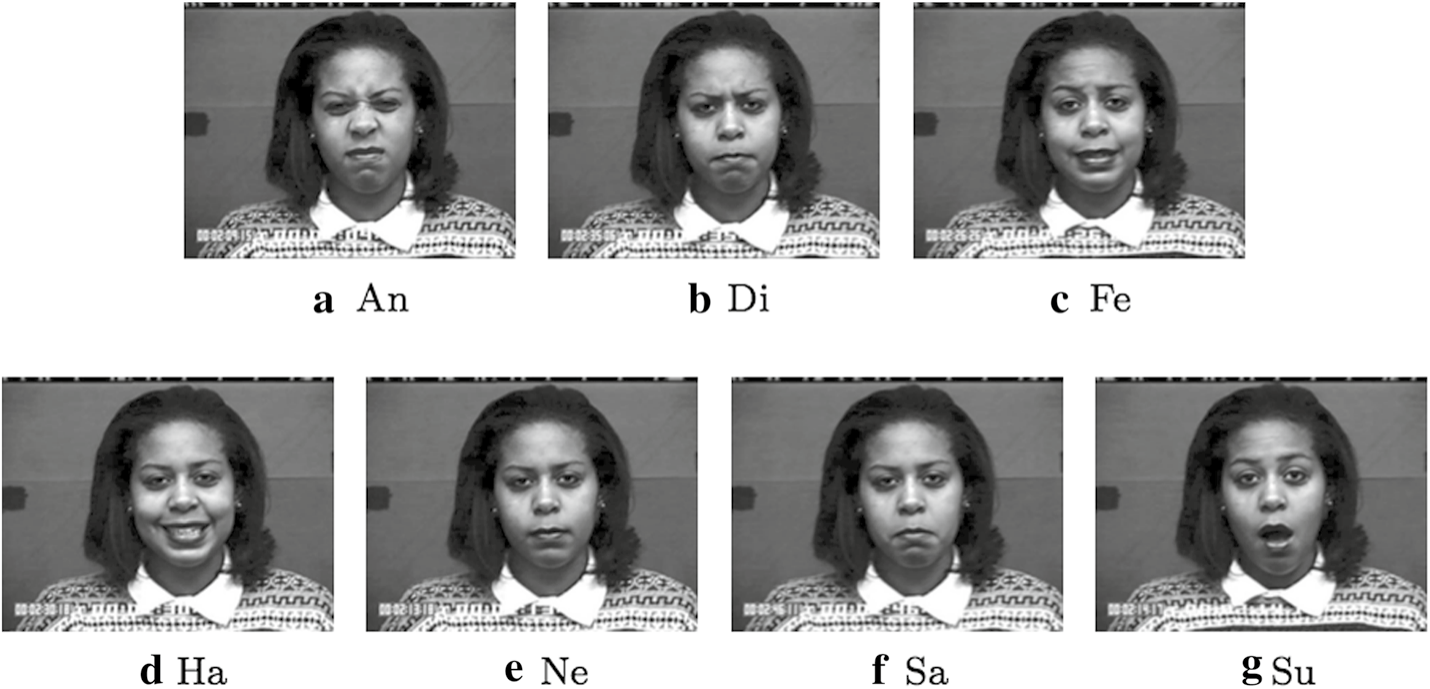
Examples of expressions for the CK+ dataset. *An* Anger, *Di* Disgusted, *Fe* Fearful, *Ha* Happy, *Ne* Neutral, *Sa* Sad, *Su* Surprised

**Fig. 5:**
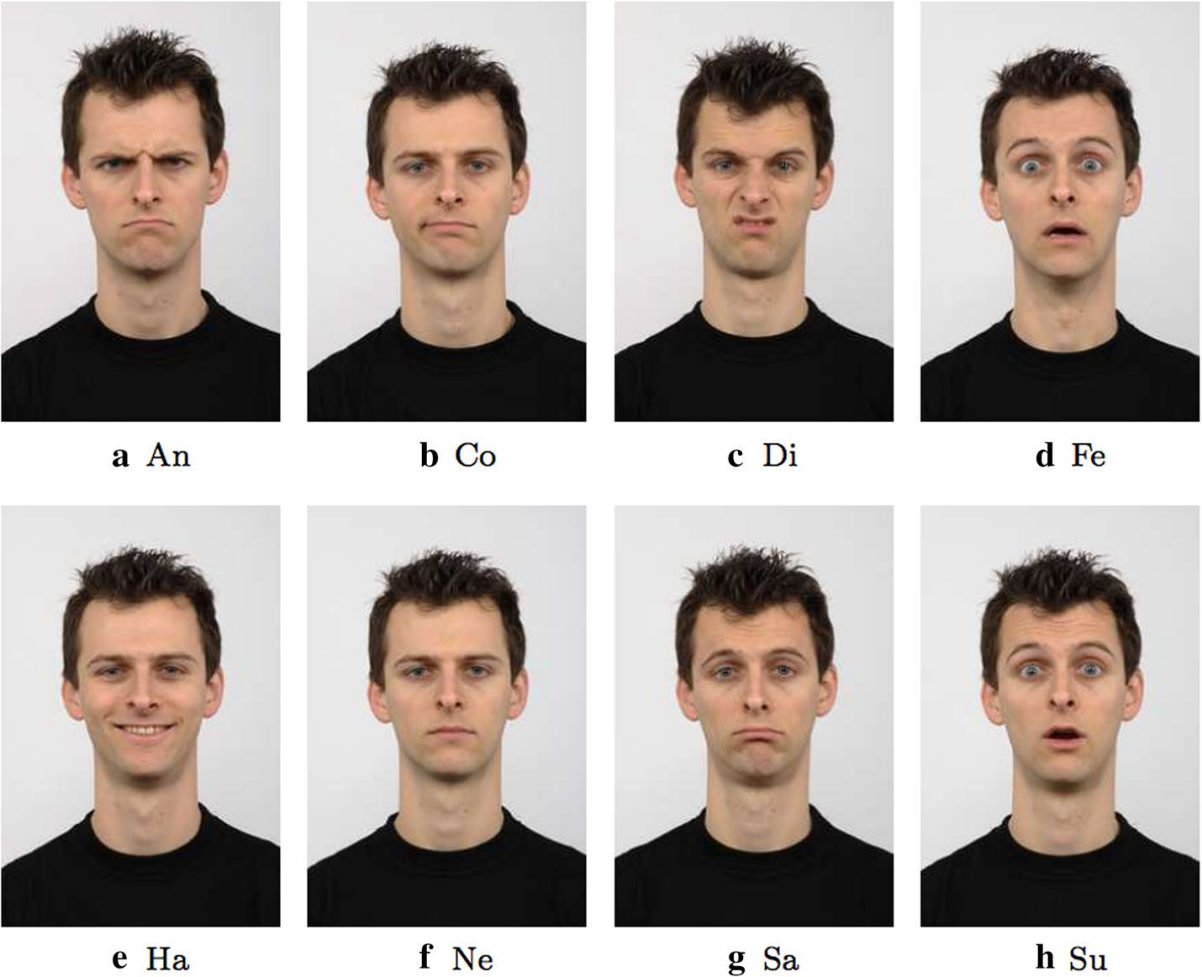
Examples of expressions for the RBD dataset. *An* Anger, *Co* Contemptuous, *Di* Disgusted, *Fe* Fearful, *Ha* Happy, *Ne* Neutral, *Sa* Sad, *Su* Surprised

In order to extract, from the available sequences, a balanced (i.e. quite the same number of instances for each considered expression) subset of images containing expressive faces, the following images were selected: the last image for the sequences related to the expression of anger, disgust and happiness; the last image for the first 68 sequences related to expression of surprise; the last and the fourth from the last images for the sequences related to the expressions of fear and sadness. At the end, a subset of 347 images was obtained with the following distribution among the considered classes of expressions: anger (45), disgust (59), fear (50), happiness (69), sadness (56) and surprise (68). An additional configuration of the previous subset was also introduced in order to test the performance with 7 classes and in this case 60 facial images, with neutral expression, were added to the aforementioned ones. In the last years a new dataset for FER analysis, named as Radboud Faces Database (RFD) Langner et al. (2010), has been introduced. The dataset contains images of 67 subjects performing 8 facial expressions (anger, disgust, fear, happiness, contemptuous, sadness, surprise and neutral) with 3 gaze directions and 5 different face orientations. The frontal facial image, with frontal gaze direction, was selected for each subject. In this way 2 subsets were obtained: the first one with 7 expressions (anger, contemptuous, disgusted, fearful, happy, sad, surprised) for a total of 469 images and the second one where 67 instances of the neutral expression were added to the previous ones (for a total of 536 images). In Figs. 4 and 5 some examples in the considered subsets of images are reported.

## Experimental results: phase 1

This section reports, in the first subsection, the procedure by which the parameters of the HOG descriptor were optimized to get the best FER performance on the Chon-Kanade (CK+) dataset with 6-expressions. In the successive subsection, the comparison of the optimized pipeline with alternative appearance features descriptors is then detailed and, in the last subsection, the comparison with related techniques in the state of the art is finally reported. All the results in this section are referred to single images and consequentially have been carried out by neglecting the time window analysis.

### HOG parameters optimization

**Fig. 6:**
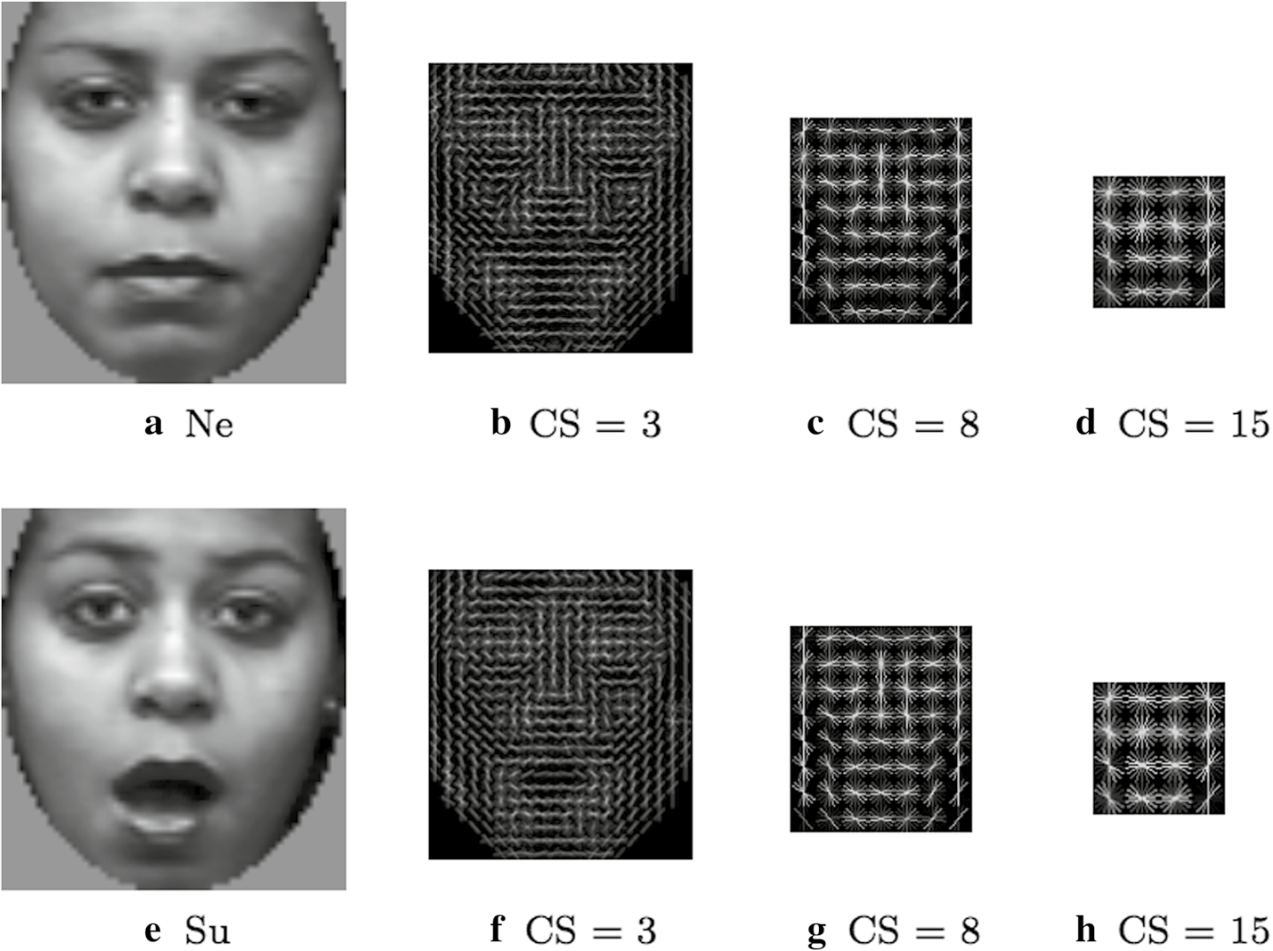
Examples of HOG (9 orientations) processing on registered face images (*Ne* Neutral, *Su* Surprised.). CS is the cell size of the processed images

This subsection is aimed at selecting the optimal values for the internal HOG parameters, i.e. the best configuration to capture the most discriminative information for the FER problem. More specifically, the average recall value has been employed for performance evaluation. This choice had been driven looking at the most recent works (as Happy and Routray 2015) where average recall is employed as the main performance value for comparison among different methods. HOG descriptor is characterized by two main parameters, the cell size and the number of orientation bins. Cell size represents the dimension of the patch involved in the single histogram computation. The importance of this parameter has been highlighted in Déniz et al. (2011) where the same grid like HOG extraction was exploited and the fusion of HOG descriptors at different scales has been used to effectively address the face recognition issue. Using a large cell size, the appearance information of a significant region of the facial image is squeezed into a single cell histogram and then some details, useful for subsequent classification, can be lost. On the other hand, with a small cell size, high resolution analysis can be carried out but, in this way, the discrimination between useful and useless extracted details is demanded to the classifier that could be unable to perform this additional task in the best way. The number of orientation bins refers instead to the quantization levels of the gradient information. A low number of orientations could lead to some loss of information and a consequent reduction in FER performance. Vice versa, a high number of quantization levels could spread-out the information along the bins, decreasing the FER performance as well. For these reasons, the choice of these parameters has to be carefully carried out by taking into consideration the goal to be reached in a particular application context. How this choice was made for FER purposes is described below. First, with regard to the cell size, a qualitative assessment can be made: in Fig. 6 the registered versions of a neutral and a surprised face expression are shown with the related processing outcomes obtained by HOG descriptor with a fixed number of 8 orientations and different values of cell size (3, 8 and 15 pixels). It is quite evident that the case with cell size of 15 pixels led to a loss of information: no correspondences between facial traits and HOG histogram can be accomplished since the accumulation of orientations was related to a large image region. On the contrary, the use of a small cell size (3 pixel) produced a very crowded distribution of the bins and then the information cannot be adequately encoded. So far, from Fig. 6, could be deduced that the most discriminative representation is given, instead, by the use of a middle cell size (in the examples 8 pixels).

The above qualitative evaluation can be also strengthened by a quantitative analysis of the FER performance sensitivity to both cell size and number of orientation bins. To perform this evaluation, the proposed algorithmic pipeline was tested using a 10-fold cross validation with 12 possible values of the cell size (from 4 to 15 pixels) and different number of orientation bins (3, 5, 7, 9, 12, 15 and 55).

**Fig. 7:**
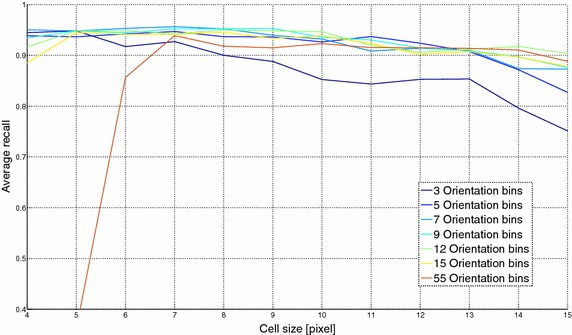
FER results using different cell sizes and number of orientation bins for the HOG descriptor: the x-axis reports the cell size in pixel and the y-axis refers to the average recall percentage

FER results, for different numbers of orientation bins, are graphically reported onto the y-axis in Fig. 7 where the x-axis reports the cell size. From Fig. 7 it is possible to infer that a cell size of 7 pixels led to the best FER performance. Concerning the choice of the number of orientations, the best results were obtained with value set to 7 even if also with 9 or 12 orientations the FER performance did not change significantly.

Choosing the optimal parameters configuration (cell size of 7 pixels and 7 orientation bins), the proposed pipeline is able to correctly classify the images, supplied as input during the 10-fold cross validation process, with average performance that can been numerically expressed with a recall of 95.9 %, a precision of 95.8 %, an accuracy of 98.9 % and a F-score of 95.8 %.

### Is HOG descriptor the best choice?

Given the used pipeline, this subsection tries to give experimental evidence of the superiority of HOG descriptor compared to three commonly used descriptors of local appearance: Local Binary Pattern (LBP) Da and Sang (2009), Spatial Weber’s Local Descriptor (SWLD) Chen et al. (2010); Ullah et al. 2012) and Compound Local Binary Pattern (CLBP) Ahmed et al. (2011). Moreover, a computational cost analysis is presented in order to have an overview also from this prospective.

More specifically, LBP is a local feature descriptor widely utilized to describe texture appearance in pattern recognition problems. The original LBP assigns a label to each pixel. Each pixel is used as a threshold and compared with its neighbors. If the neighbor is higher it takes value 1 otherwise it takes value 0. Finally, the thresholded neighbor pixel values are concatenated and considered as a binary number that becomes the label for the central pixel. To account for the spatial information, the image is then divided into sub-regions. LBP is applied to each sub-region and a histogram of L bins is generated from the pixel labels.

SWLD is the spatial version of WLD, a robust descriptor inspired by the psychological Weber’s law (Jain 1989). It is based on the fact that the human perception of a pattern depends on the amount of change in intensity of the stimulus as well as on the original stimulus intensity. The WLD descriptor consist of two components: differential excitation (to detect the local salient patterns) and gradient orientations.

Finally, the CLBP is an extension of the LBP descriptor. Compared to LBP, CLBP encodes the sign of the differences between the center pixel and its P neighbors as well as the magnitude of the differences.

For all the aforementioned descriptors, a preliminary parameters optimization procedure was done. After that, the LBP and CLBP with a radius of 1 pixel and 8 neighbours over a 5 $\times$ 5 grid and the SWLD with values of parameters T, M, S set to 8, 4 and 4 over a 4 $\times$ 4 grid were used.

Table 1 reports the average FER performance along the 10-fold cross validation on the Chon-Kanade (CK+) dataset with 6-expressions: it is possible to observe that none of the comparing techniques (LBP 91.7 %, CLBP 92.3 %, SWLD 86.5 %) reached the FER performance of the HOG descriptor (95.8 %) and these results confirmed that the edges and shapes modeling performed by HOG is more suitable to describe the facial expression if compared with the texture oriented descriptors that are unable to describe the facial deformation among different expressions.

**Table 1:** Performance comparison of HOG descriptor respect to LBP, CLBP and SWLD descriptors

LBP	CLBP	SWLD	HOG
91.7	92.3	86.5	*95.8*

Italic value indicates the best result

**Fig. 8:**
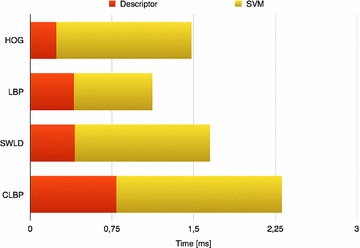
*Bar* diagram of processing time for descriptor computation and SVM prediction stages respect to LBP, CLBP and SWLD descriptors

The computational cost prospective has to be discussed too. The values (processing time for the descriptor computation and the SVM prediction), reported in Table 2 and illustrated in the bar diagram of Fig. 8, have been computed considering an average of 500 frames employing an Intel i7 processor class based Linux machine. The analysis of these results shows as the LBP descriptor allows to save about 0.4 ms with respect to the HOG one. Nevertheless, the processing time for the face detection and registration step (41, 9 ms) is 2 magnitude greater than the discussed times making this advantage quite negligible.

**Table 2:** Processing time for the detection, descriptor computation and SVM prediction stages

	Detection	Descriptor	SVM	Total time
HOG	41.9	0.24	1.24	43.38
LBP	41.9	0.40	0.72	43.02
SWLD	41.9	0.41	1.24	43.55
CLBP	41.9	0.79	1.52	44.21

All the values are expressed in ms

### Comparison with the state of the art

In this section the achieved results are compared with those of the leading State-of-the-Art FER solutions. Differently from other research fields, in the FER one there is not a shared dataset to be used as benchmark for a fair evaluation of different algorithms. In order to avoid errors introduced by the re-implementation of each method, most of the works in the literature refer to CK+ dataset that, unfortunately, is dramatically unbalanced and, for this reason, it requires a selection of a subset of available expression occurrences before to be used. How this selection has to be performed is not well stated and then the reported comparisons are always biased by this important drawback. In addition, there is not a standardised evaluation procedure even if most of the works make use of a cross-validation procedure and then represent the recognition performance by means of confusion matrices. In order to accomplish this crucial task, in this paper, the approach implemented in the most up-to-date works for FER recognition is then used (Happy and Routray 2015), i.e. a CK+ subset of observations was selected and then a k-fold cross validation was used to fill the confusion matrix. In this way, the performance of the comparing approaches can be extracted from the relative papers avoiding to affect them by implementation issues. To be fairest as possible, for most of the sequences in the dataset, we retained only one image (we experimentally proved that choosing more than one image the overall performance increase). For balancing reasons, for a few sequences also the fourth image from the last one was retained and, in this way, the largest possible subset of images (in respecting a reasonable balancing among categories) was build. Choosing the fourth from the last image introduces less correlation then the third from the last or the second from the last.

Table 3 reports the comparison results demonstrating that the proposed approach gave the best average recognition rate. In particular, it is worth noting that the performance achieved by the approach under investigation exceed also those of the recent work in Happy and Routray (2015) that represents the reference point for the FER problem. A deeper analysis of the Table 3 evidences that the proposed method suffers more than competitors to recognize the expression of disgust. This drawback could be due to the fact that, while performing this expression, the facial muscles shape is quite similar to that of the expression of anger (see Figs. 4, 5) hence the edge analysis performed by HOG, sometimes, cannot be able to bring to light differences as other approaches based on texture analysis can instead highlight. However, this is a limitation only for the recognition of the expression of disgust since for all the remaining expressions the FER performance of the proposed method largely exceed those of the comparing methods highlighting that the analysis of the edges is the best method to recognize facial expressions as it throws away all possible ambiguity introduced by non-edge based features.

**Table 3:** Performance comparison of our approach versus different State-of-the-Art approaches (CK+ 6 expressions)

	Uddin et al. (2009)	Poursaberi et al. (2012)	Zhong et al. (2012)	Zhang and Tjondronegoro (2011)	Happy and Routray (2015)	Proposed
An	82.5	87.1	71.4	87.1	87.8	88.6
Di	97.5	91.6	95.3	90.2	93.3	89.0
Fe	95.0	91.0	81.1	92.0	94.3	100
Ha	100	96.9	95.4	98.1	94.2	100
Sa	92.5	84.6	88.0	91.5	96.4	100
Su	92.5	91.2	98.3	100	98.5	97.4
AV	93.3	90.4	88.3	93.1	94.1	*95.8*

Italic value indicates the best result

*An* Anger, *Di* Disgusted, *Fe* Fearful, *Ha* Happy, *Sa* Sad, *Su* Surprised

## Experimental results: phase 2

This section extends the experimental evaluation of the proposed solution to other datasets of facial expressions. In particular, the next subsection reports the analysis of the HOG parameters repeated using alternatively as benchmark one of the remaining 3 datasets described in "Experimental data setup". During this experimental evaluation it was demonstrated that, given the preliminary registration of the facial images, the configuration process converged to the same values, i.e. it was independent from the considered dataset. After that, in the last subsection, the detailed FER accuracies of the proposed approach, for all the datasets, are reported using confusion matrices that are then deeply discussed.

### Generalization of the optimization of HOG parameters

**Fig. 9:**
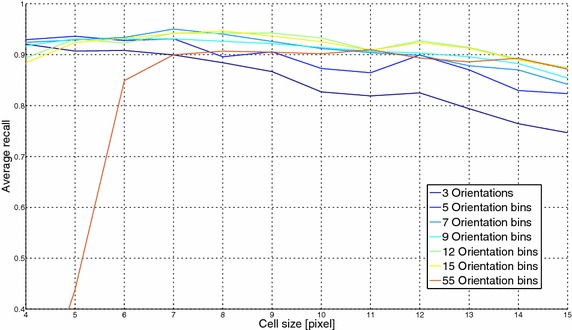
HOG optimization results (CK+ with 7 expressions): the x-axis reports the cell size in pixel and the y-axis refers to the average recall percentage

**Fig. 10:**
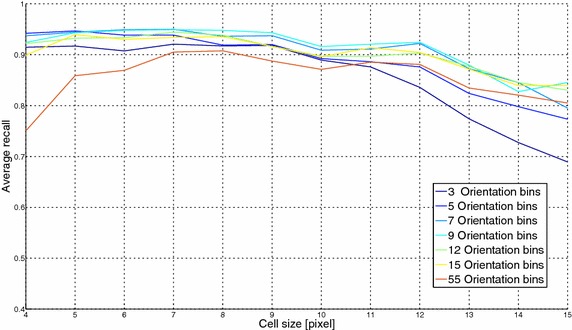
HOG optimization results (RFD with 7 expressions): the x-axis reports the cell size in pixel and the y-axis refers to the average recall percentage

**Fig. 11:**
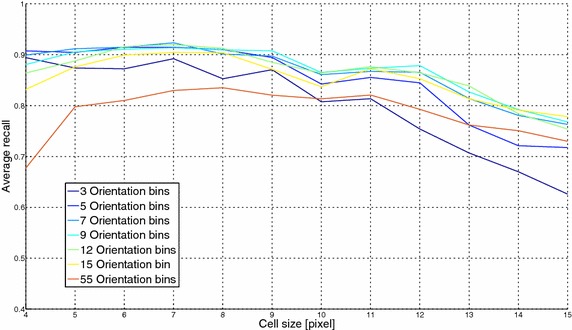
HOG optimization results (RFD with 8 expressions): the x-axis reports the cell size in pixel and the y-axis refers to the average recall percentage

In this subsection, the optimization of the HOG parameters (already carried out for the 6-expressions CK+ dataset and reported in "Experimental results: phase 1") is extended to the remaining 3 datasets described in "Experimental data setup": the CK+ with 7 expressions and the RFD with 7 and 8 expressions. This experimental step was carried out in order to verify that the best configuration of HOG parameters revealed in "Experimental results: phase 1" keeps still valid also for a different testing set. The plots relative to the 3 related additional experiments are shown in Figs. 9, 10 and 11 and they demonstrate that a cell size of 7 pixels and 7 orientation bins is again the best HOG parameters configuration to obtain the highest FER performance. This is a very important result, since it allows the proposed pipeline to be used without any initial parameter setting, even in the presence of different operative contexts. In other words, given the preliminary image registration, the FER proposed solution becomes parameter independent and then suitable to be used as a black-box.

### Confusion matrices for all the datasets

**Fig. 12:**
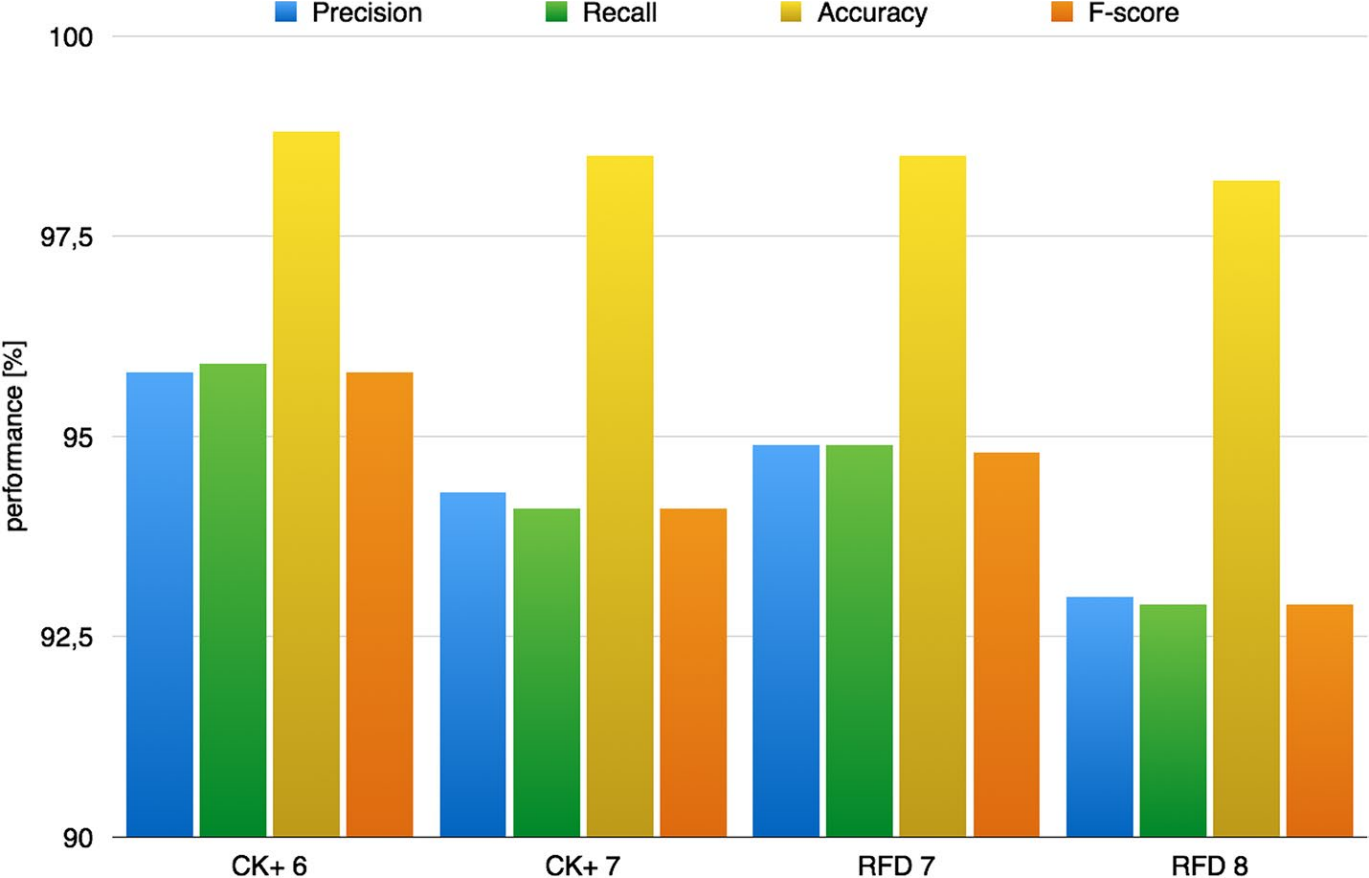
Bar diagram of average precision, recall, accuracy and F-score for the 4 tested datasets

Once established, in previous subsection, that there is a unique best configuration of the HOG parameters, the performance of the proposed approach were better analyzed. In particular, in a multi-class recognition problem, as the FER one, the use of an average performance value among all the classes could be not exhaustive since there is no possibility to inspect what is the separation level, in terms of correct classifications, among classes. To overcome this limitation, for each dataset the confusion matrices [expressed in terms of recall in order to keep coherence with other works (Happy and Routray 2015)] are then reported in Tables 4, 5, 6 and 7. This makes possible a more detailed analysis of the results that can point out the misclassification cases and the interpretation of their possible causes. Additional information is given in the bar diagram of Fig. 12 and in Table 8 where the average precision, recall, accuracy and F-score are provided for all the datasets. First of all, from the confusion tables it is possible to observe that the proposed pipeline achieved an average performance value rate over 90 % for all the tested datasets and that, as expected, its FER performance decreased when the number of classes, and consequently the problem complexity, increased. In fact, in the case of the CK+ dataset with 6 expressions, the recall was of 95.9 % whereas after the addition of the neutral expression it decreased to 94.1 %. For the RFD, with 7 expressions, the FER recall was of 94.9 % whereas the addition of the neutral class led to a value of recall of 92.9 %. These are very encouraging results considering the challenging benchmark used for testing.

**Table 4:** Performance of proposed approach (CK+ 6 expressions)

	An	Di	Fe	Ha	Sa	Su
An	88.6	4.5	2.4	0	4.5	0
Di	5.6	89.0	1.8	1.8	0	1.8
Fe	0	0	100	0	0	0
Ha	0	0	0	100	0	0
Sa	0	0	0	0	100	0
Su	1.3	0	1.3	0	0	97.4

Average performances: recall = 95.9 %, precision = 95.8 %, accuracy = 98.8 %, F-score = 95.8 %. (orientation bins = 7, cell size = 7)

*An* Anger, *Di* Disgusted,* Fe* Fearful,* Ha* Happy, *Sa* Sad, *Su* Surprised

**Table 5:** Performance of proposed approach (CK+ 7 expressions)

	Ne	An	Di	Fe	Ha	Sa	Su
Ne	89.6	1.8	0	0	0	8.6	0
An	4.4	86.8	4.4	0	0	4.4	0
Di	0	5.4	92.9	1.7	0	0	0
Fe	0	0	0	93.9	4.1	0	2.0
Ha	0	0	0	0	100	0	0
Sa	0	0	1.8	0	0	98.2	0
Su	1.3	0	0	1.3	0	0	97.4

Average performances: recall = 94.1 %, precision = 94.3 %, accuracy = 98.5 %, F-score = 94.1 %. (orientation bins = 7, cell size = 7)

*Ne* Neutral, *An* Anger, *Di* Disgusted,* Fe* Fearful,* Ha* Happy, *Sa* Sad, *Su* Surprised

**Table 6:** Performance of proposed approach (RFD 7 expressions)

	An	Co	Di	Fe	Ha	Sa	Su
An	98.4	0	0	0	0	1.6	0
Co	3.0	92.5	0	1.5	0	3.0	0
Di	0	0	97.0	0	0	1.5	1.5
Fe	0	1.5	0	94.0	0	1.5	3.0
Ha	0	0	0	0	100	0	0
Sa	6.0	1.5	1.5	7.4	0	83.6	0
Su	0	1.5	0	0	0	0	98.5

Average performances: recall = 94.9 %, precision = 94.9 %, accuracy = 98.5 %, F-score = 94.8 %. (orientation bins = 7, cell size = 7)

*An* Anger, *Co* Contemptous, *Di* Disgusted, *Fe* Fearful, *Ha* Happy, *Sa* Sad, *Su* Surprised

**Table 7:** Performance of proposed approach (RFD 8 expressions)

	An	Co	Di	Fe	Ha	Ne	Sa	Su
An	97.0	0	0	0	0	1.5	1.5	0
Co	1.5	88.1	0	0	0	7.5	2.9	0
Di	0	1.5	98.5	0	0	0	0	0
Fe	0	0	0	92.5	0	3.0	0	4.5
Ha	0	1.5	0	0	98.5	0	0	0
Ne	0	10.4	0	0	0	86.6	3.0	0
Sa	7.4	3.0	0	3.0	0	1.5	85.1	0
Su	0	1.5	0	0	0	1.5	0	97.0

Average performances: recall = 92.9 %, precision = 93.0 %, accuracy = 98.2 %, F-score = 92.9 %. (orientation bins = 7, cell size = 7)

*An* Anger, *Co* Contemptous, *Di* Disgusted, *Fe* Fearful, *Ha* Happy, *Ne* Neutral, *Sa* Sad, *Su* Surprised

Looking at the bar diagram in Fig. 12 and at the values in Table 8 it is possible to observe also the other considered metrics, as precision, accuracy and F-score, had the quite same behavior of the recall one among the different datasets.

**Table 8:** Performance of proposed approach among different datasets (CK+ 6 expressions, CK+ 7 expressions, RFD 7 expressions, RFD 8 expressions)

	Pecision	Recall	Accuracy	F-score
CK+ 6	95.8	95.9	98.8	95.8
CK+ 7	94.3	94.1	98.5	94.1
RFD 7	94.9	94.9	98.5	94.8
RFD 8	93.0	92.9	98.2	92.9

Going into a more detailed analysis, Tables 4 and 5 highlight an ambiguity between anger, disgusted and sad expressions. This becomes quite reasonable if the examples in Figs. 4 and 5 are observed: for all the aforementioned expressions, strict lips and low position of eyebrows are in fact very similar, in both location and appearance. For the same reasons, the neutral expressions introduced some misclassifications in Tables 5 and 7 whereas the contemptuous expression was sometimes classified as neutral or sad one. Similarly, the sad expression experimented some erroneous classification in the anger face expression due to the strict lips and low position of eyebrows that are very similar for the two expressions. Finally, the happy expression is the most insensitive to ambiguities and reached the 100 % of classification in all the tests (except for the RFD with 6 expressions).

## Experimental results: phase 3

The experiments reported in previous sections were relative to the recognition of facial expressions in a single image containing a clearly defined expression. In common application contexts, the automatic systems have to perform FER by analyzing image sequences in which not all the images contain a clear expression, or where there are transitions between expressions. This section aims, thus, at analyzing the behavior of the proposed pipeline, in its whole proposed form, when applied to video sequences. To this end the experiments of this phase were conducted in two steps: a quantitative accuracy evaluation using video sequences of the CK+ dataset in the first step; a qualitative accuracy evaluation using on-line continuous video streaming in the second step. In both steps the system has been equipped with the model trained on the CK+ dataset with 7 expressions employed in the previous experiments.

In the first step, the quantitative performance evaluation of the capability to recognize the expressions embedded into the video sequences of the CK+ dataset is reported. CK+ sequences start with a neutral face expression that evolves to the expressive one; the number of frames, for a particular expression, changes for every subject. In this case the time window has been customized to manage this specific expression change. More specifically, a sequence *i* was considered correctly recognized if in the first $m_1$ frames there exist at least $n_1$ images classified as containing the neutral expression and, at the same time, in the last $m_2$ frames there exist at least $n_2$ images classified as containing the expression with which the sequence is labeled. In the experiments, the following setting was used: $n_1=1$, $m_1=3$, $n_2=4$, $m_2=5$ and, over the 327 labeled sequences, the percentage of correct classification was of $72.2~\%$.

In Fig . 13 two examples of correct classification of CK+ image sequences are reported. In the first one, both the first 3 frames and the last 5 frames were correctly classified whereas in the second one an error occurred in the second frame but that was filtered by the overall decision rule.

**Fig. 13:**
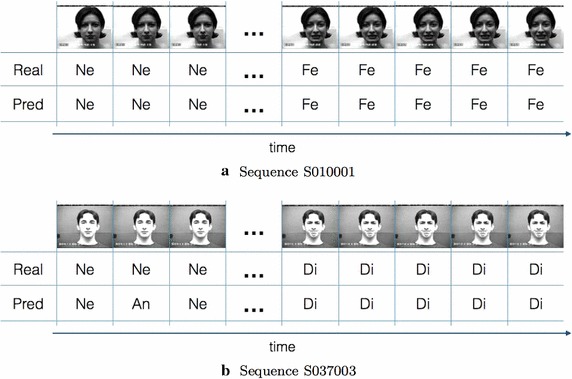
Examples of expression recognition in CK+ sequences: **a** from Neutral to Fearful and **b** from Neutral to Disgusted. For each sequence the first 3 and the last 5 frames are reported i.e. the frames containing the labelled expressions. The *first row* reports the facial images, the *second row* shows the ground-truth labels and finally, in the *third row*, the predicted expressions are pointed out

**Fig. 14:**
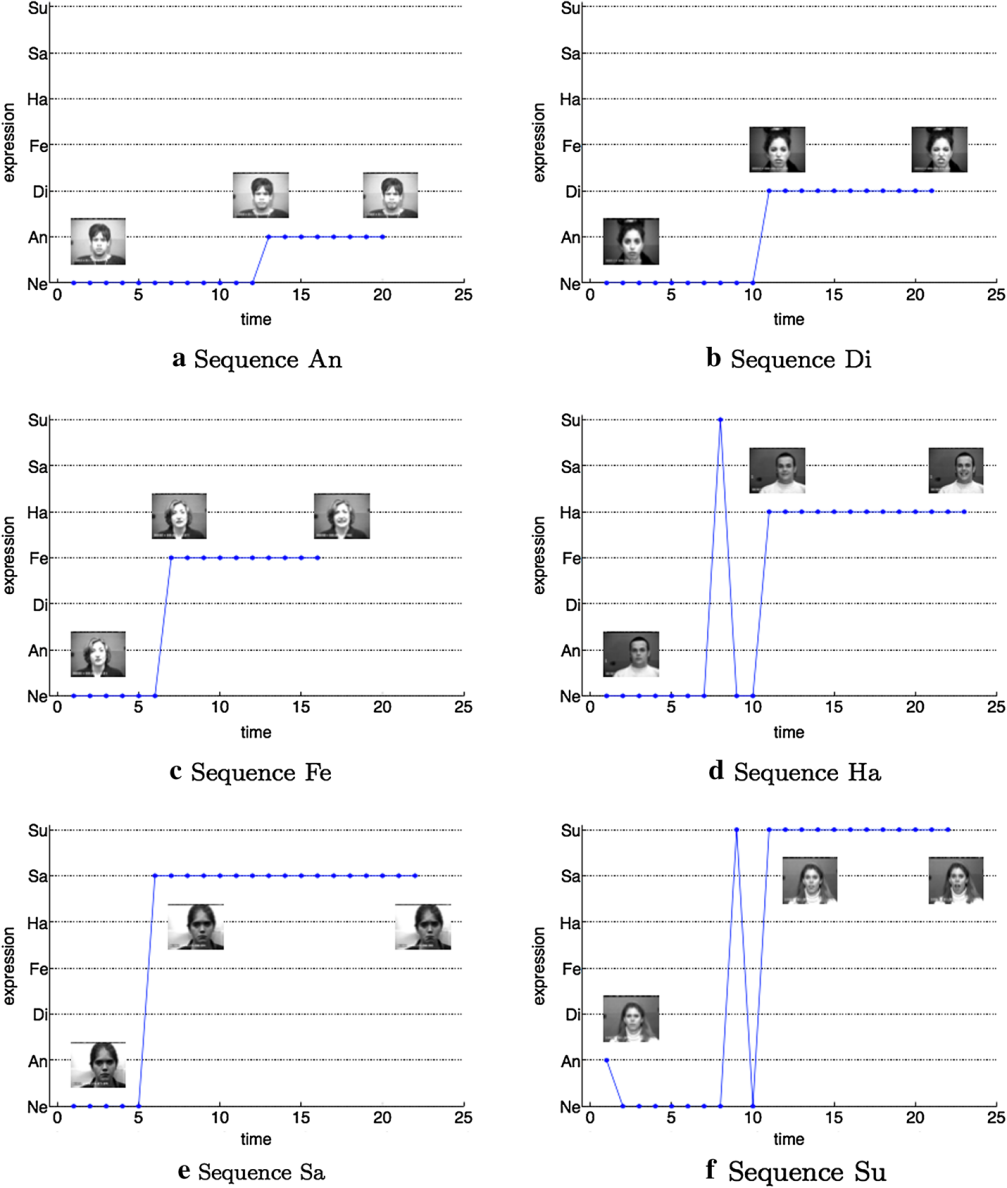
Plot of detected emotions on 6 examples of CK+ sequences: **a** from Neutral to Angry, **b** from Neutral to Disgusted, **c** from Neutral to Fearful, **d** from Neutral to Happy, **e** from Neutral to Sad, **f** from Neutral to Surprised. The *x* axis is representative of the temporal variations of the expression (from neutral to expressive); the *y* axis shows the predicted expression (*Ne* Neutral, *An* Anger, *Di* Disgusted, *Fe* Fearful, *Ha* Happy, *Sa* Sad, *Su* Surprised.). For each plot the starting image, the image after the expression prediction change and the final expression are reported

In order to get evidence of how the system works on the whole image sequences, in Fig. 14 the system outcomes on 6 sequences of the CK+ dataset are plotted along time. It can be observed that in the most of cases a sharp prediction change occurred along the expression mutation whereas sometimes the prediction is unstable due to the uncertainty of the system over the transition frames.

Finally, in the second experimental step, an on-line test, exploiting the whole process proposed in "Methodology overview", was performed using an ARM Cortex A-15 class processor (2.3 Ghz) embedded system equipped with a 640 $\times$ 480 pixels webcam. Subjects of different gender and age have been involved in the experiments. More specifically, to all subjects were asked to sit in front of the camera and to randomly perform some of the aforementioned expressions. It is worth noting that no constraints, about the transition from the neutral expression to the expressive one, have been introduced leaving the user free to perform every possible transition among different expressions.

**Fig. 15:**
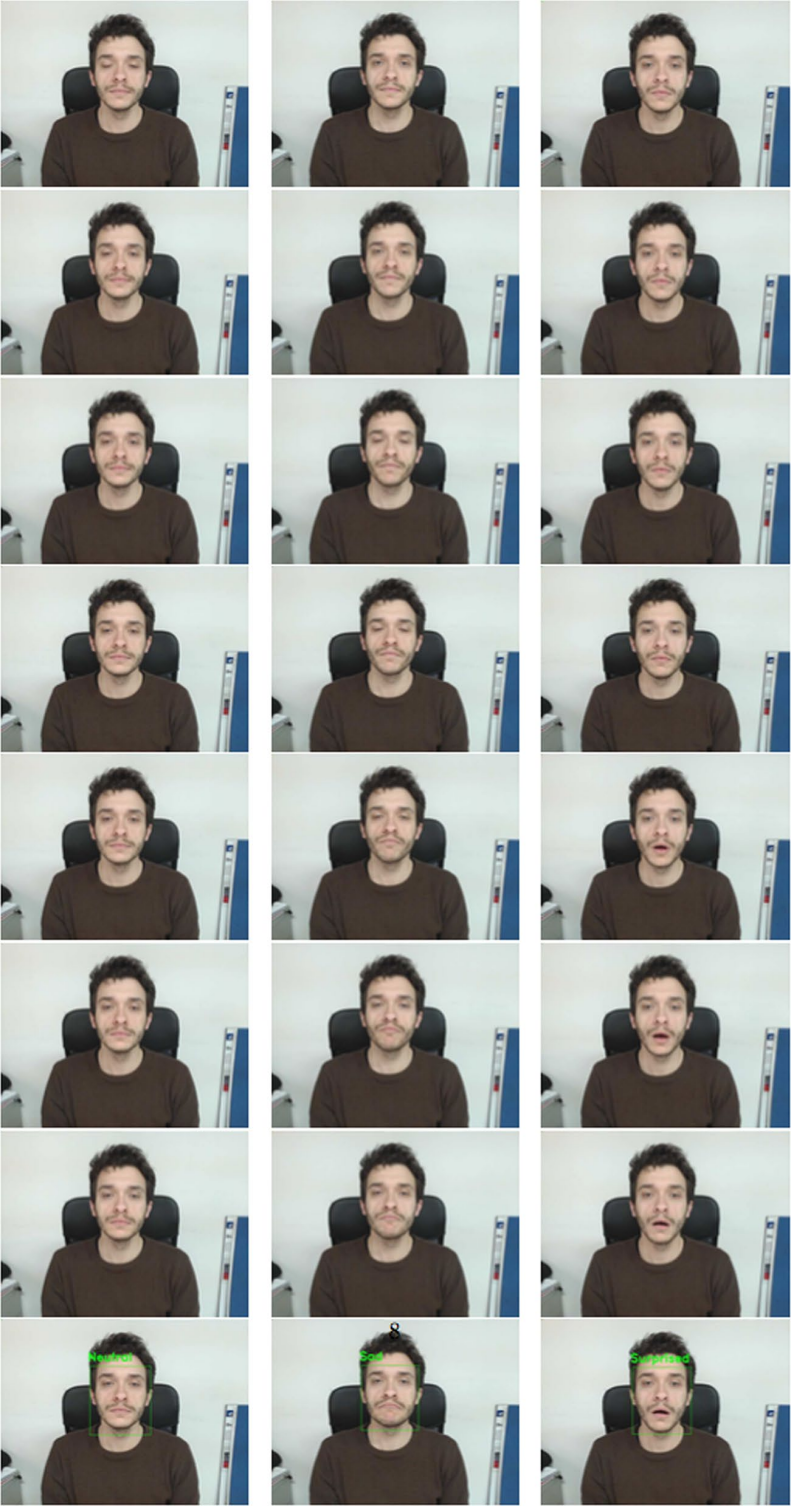
Examples of expression detection performed by the proposed system: the expression evolves over time (from the *top to the bottom*); once the decision making rule is satisfied, the out-coming prediction is printed out. From *left to right* the neutral, sad and surprised expressions are shown

The system evaluated in this step, from a qualitative point of view, exhibited a good capacity to recognize all the emotions performed by the users with a quite low presence of false positives, thanks to the filtering performed by the temporal windows approach. Some examples of the system output are reported in Fig. 15.

Finally, it is worth noting as the proposed approach is able to reach a processing rate of 7 fps that is a notable value taking into account the limited computational resources of the employed embedded system. By our experience, such a frame processing rate is sufficient for considering the proposed system as suitable for real time applications oriented to implement a natural human–machine interaction.

**Fig. 16:**
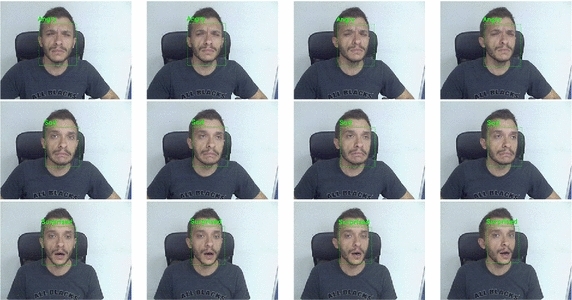
Examples of expression detection with non-frontal faces. The qualitative tests show that, for an angle between −30 and 30 degrees, the detector is able to correctly register the face and the classifier is enough robust to guarantee classification performance comparable with the frontal face case

As an additional experiment the robustness to non-frontal face poses was tested. As expected, the detector was able to work in a range of [-30,30] degrees of variation with respect to the frontal view. All the expressions in the detected faces were correctly classified by the system demonstrating its capability to hold very good FER performance (comparable with those obtained in case of frontal faces) even in case of non-frontal facial views. Figure 16 shows some examples of classification of expressions when different degrees of facial orientation were experienced.

## Conclusions

In this paper a comprehensive study of how the HOG descriptor could be effectively exploited for facial expression recognition purposes has been carried out. In order to perform the above mentioned investigation, a pipeline, commonly used in FER analysis and equipped with an HOG descriptor, has been used as baseline. Then, the classification performance, on a well known FER dataset, has been analyzed as a function of the HOG parameters setting (cell size and number of orientation bins). Results highlighted a configuration of HOG parameters able to fit the specific aspects of facial expressions that allows a high classification performance capable to overcome the performances of the leading state of the art approaches. In order to prove that the achieved HOG configuration is general, i.e. it does not depend on the input data, the pipeline was tested on three additional datasets. As a final step, experiments on video sequences gave evidence that the proposed approach is also suitable to be used in real-world application contexts.

Future works will deal with two main aspects. On the one hand the system will be tested in the field of assistive technologies. In particular, a humanoid social robot will be equipped with the proposed FER solution in order to acquire awareness about the emotional state of the interacting user and consequently to react with a specific behavior. On the other hand a more wide analysis of the influence of non frontal face effect will be done and the effectiveness of face frontalization algorithm Hassner et al. (2014) will be tested.
